# Is competition good or bad for the price, quantity, and quality of bank lending?

**DOI:** 10.1371/journal.pone.0287002

**Published:** 2023-08-10

**Authors:** Japan Huynh

**Affiliations:** Faculty of Finance and Banking, Ho Chi Minh City Open University, Ho Chi Minh City, Vietnam; Alexandru Ioan Cuza University of Iasi, ROMANIA

## Abstract

This paper extends the existing literature by examining an important channel through which bank competition could drive the real economy by comprehensively influencing bank lending in three components–price, volume, and quality. For the measurement of bank competition, we build a series of different structural (concentration indicators) and non-structural (Lerner, Boone, and Panzar-Rose H-statistic indexes) measures, given that the reliance on solely one individual measure could lead to a misleading conclusion. Through a sample of commercial banks during 2007–2021 in a single Vietnamese banking market, we find a decline in bank loan growth and a rise in credit risk under the pressure of high competition. With respect to the association between bank competition and the price of credit, our empirical evidence is mixed based on different measures to analyze the banking market structure. Our findings support the view that greater competition results in a less proliferated banking sector with riskier assets. We also confirm that these findings are robust to additional tests, including employing alternative measures of bank lending dimensions and market structure, removing the periods of the financial crisis and the COVID-19 pandemic, and changing the empirical estimation technique. In addition, our deeper analysis reveals that the adverse impact of bank competition on lending, shown by reduced credit supply and increased credit risk, is less pronounced for banks with a higher degree of income diversification. This result suggests that bank diversification may protect the quantity and quality of bank lending from the detrimental competition effect.

## Introduction

In the banking sector, competition is encouraged due to its benefits. The traditional view is that increased banking competition is associated with a drop in lending rates, which is an essential driver of increased business investment and improved economic growth [1, 2]. In competitive markets, the allocation of capital resources is efficient, and it is less likely for a few large banks to dominate the market, manipulate the cost of credit, and restrict firms’ access to credit. Banking competition can offer welfare gains by wiping out monopoly rents and reducing cost inefficiencies, thereby leading to better access to financial services and improved financial stability [3]. Furthermore, efforts to increase competition are motivated by the evidence that this may help to prevent the likelihood of a crisis and extend the time to a crisis [4]. As a result, policymakers understand the value of bank competition and then try to establish policies that stimulate competition, such as interest rate liberalization, banking deregulation, and eliminating entry barriers [5].

However, despite its numerous benefits, one has still been concerned about the negative aspects of high competition in the banking sector. The largest concern is based on the competition fragility view [6], implying that greater competition may damage bank prudence through reduced profits, thereby raising risk-taking incentives of incumbent banks and posing a threat to the safety and soundness of the banking system. Also, increased competition may discourage banks from investing in lending relationships and negatively influence enterprises’ access to finance [7]. Besides, as demonstrated by the literature on the structure of the banking sector, more competition might enhance the probability of bank failure [8], and a banking sector with a high level of concentration is more likely to reduce a chance of a crisis [9].

This paper adds to the above arguments by examining competition and bank lending. Theoretical arguments have suggested various ways through which banking competition may influence the costs of credit services, the access to finance, and the stability of financial institutions. Most prior studies focus on one single aspect of bank lending when analyzing the competition effect, for example, the credit price [10, 11], the credit volume [12, 13], and the credit risk [14, 15]. However, none of them comprehensively addresses all three critical dimensions of bank lending in the relationship with competition simultaneously. The literature claims that bank concentration results in more loan defaults since higher lending rates in consolidated systems place a significant financial burden on borrowers [16]. However, no empirical study has been performed to test the mechanism of increased risk incentives through interest rates. Hence, this paper fills in the literature gap by explicitly analyzing the influences of banking market structure on lending activities based on their three components–prices, volume, and quality. Each of these dimensions may influence banks themselves and the real economy in distinct ways. Importantly, we desire to explore whether banking competition can contribute to the real economy via the development of the banking system, shown by increased loan supply at low prices with low risks.

We empirically conduct our analysis employing a sample of commercial banks in Vietnam over the period 2007–2021. A critical issue that emerges in our estimation is the reliable construction of bank competition measures. The literature segment on the measurement of bank competition has been well established, and multiple measures have been employed in this regard. These measures comprise (i) the k-bank market concentration ratio, (ii) the Herfindahl-Hirschman concentration index, (iii) the Boone indicator, (iv) the Lerner index, and (v) the H-statistic indicator. As suggested by previous scholars, it is far better to utilize multiple proxies instead of relying on one single measure of bank competition as different competition proxies may look into different aspects of the banking market structure and the use of only one single competition measure could offer misleading information [17, 18]. Additionally, another key objective of this paper is to examine whether or not income diversification drives the competition-lending nexus. Diversification is not only an important strategy that banks apply when running their business, but it also serves well as a risk absorber that helps banks mitigate the threat of detrimental shocks [19]. Under the pressure of competition, we could expect it to cause such shocks to banks. Motivated by these arguments, we perform further analysis looking at the moderating role of income diversification, given the context that scarce attention has been paid to this issue thus far in the literature.

We also need to clarify some motivations for the selection of Vietnam as a research sample. In fact, this small and open country introduces various favorable conditions to conduct our research issues. The banking sector of Vietnam has been the main fund supplier to fuel the economic sectors [20]. In other words, the significance of lending segments in the Vietnamese banking sector is explicitly pronounced. Vietnam officially joined the World Trade Organization (WTO) in 2007, and due to this catalyst, the banking system in this country has exhibited major reforms. For example, foreign bank penetration was encouraged and thus led to significant changes in the banking market structure, where state-owned banks reduced their dominant positions [21]. Other small private banks had to compete fiercely with each other and foreign counterparts. The high-standard risk management program was activated across the banking system, and banks were also suggested to be listed on the stock exchanges. Besides, in the period of the comprehensive reform of the banking industry since 2015, as a consequence of the explosion of bad debts and the inefficiencies of weak banks, the orientation to consolidate banks and encourage banks to diversify has been particularly emphasized by the State Bank of Vietnam (SBV). This context demonstrates that studying the impact of competition on bank lending could be highly relevant and thereby provide practical implications for Vietnam as well as other markets with similar backgrounds.

Our paper expands the literature stream on banking market structure by conducting the first analysis of how competition in the banking system simultaneously influences three essential aspects of bank lending: price, quantity, and quality. Previous authors examine the competition-lending nexus with their separate interest in one single dimension of bank lending, while the coverage of various aspects of bank lending in this paper is appropriate to draw a comprehensive picture of the topic. In doing this, we also complement the literature in some ways. Most importantly, though prior scholars favor one particular methodology to capture bank competition over others, there is generally no consensus among them with regard to the best competition measure. In this paper we consider a wide range of competition measures, including the top-bank concentration ratio, the Herfindahl-Hirschman index (HHI), the Lerner index, the Panzar-Rosse H-statistic index, and the Boone indicator. Furthermore, the mixed results of regressions imply that how scholars define bank competition may greatly influence their findings; thus, using only one type of competition measurement is extremely risky for empirical analysis and should be avoided at all costs [17, 18]. The paper also contributes to the literature by exploring the significance of bank business models for the linkage between competition and bank lending activities. Although there have been studies contending that competition is the main reason banks adopt diversification strategies [22], as well as numerous papers on the effects of competition on separate aspects of lending (see our review in section 2), our work is the first to reveal the moderating role of bank diversification in the competition-lending nexus. Shedding light on this issue will be helpful to offer policy implications in emerging economies where banks have been operating under tremendous pressure from competition in the era of increased deregulations and encouraged diversification.

The remainder of this paper may flow as follows. Section 2 reviews the theoretical and empirical predictions that motivate our analysis and help develop our hypotheses relating to: (i) bank competition and credit price, (ii) bank competition and credit quantity, and (iii) bank competition and credit quality. Section 3 describes the methodology and data we use to obtain research results–subsequently reported and discussed in Section 4 along with robustness investigation. We finally conclude our study with some relevant implications in Section 5.

## Literature review and hypotheses development

### Bank competition and credit prices

Fundamentally, the primary motivation for banking competition is attributed to the booster of economic growth via the channel of lower prices. Concretely, banks with greater market power operating in highly concentrated systems may face less competition, and thereby they can charge higher interest rates to enterprises thanks to oligopolistic power (16]. Following this line of argument, banking competition is desirable as a higher degree of competition in the banking system may help to result in lower lending rates, which is beneficial to the real economy.

However, from the empirical perspective, the recent empirical evidence in the banking literature on the link between competition and credit price is limited and mixed. While some authors reveal lower lending rates amid increased bank competition [23], others indicate that market power helps to cut the cost of financing for firms [10, 11]. Notably, these papers focus on financial institutions in advanced economies (i.e., Japan, EU countries, and the US) but not emerging markets like Vietnam.

Overall, though empirical studies with different approaches using various samples suggest ambiguous implications for credit prices under the pressure of competition, we still expect the benefit of competition in reducing interest rates, as commonly suggested by the theoretical literature. Accordingly, we propose our testable hypothesis as follows:

Hypothesis 1. Higher competition reduces the bank’s credit prices.

### Bank competition and credit quantity

Banks may modify the availability of credit supply in response to changes in market structure in several ways. One stand of existing literature suggests that banks enjoying high market power in concentrated markets are better able to build close lending relationships with their customers [24]. These banks may benefit from reduced information asymmetries, thereby resulting in enhanced lending availability in banking environments that are under the control of a few dominant banks [25]. In addition, the literature also links greater market power with better access for banks to alternative funding sources. This implies that it is easier for dominant banks to obtain funds in the financial markets. As a result, they possess a more remarkable ability to grant loans to the economy [26].

Another strand of arguments suggests a competing hypothesis. As a common practice, market contestability may lead to a downward impact on lending rates, thereby supporting enterprises to approach finance more easily and cheaply [16]. Furthermore, banks are more willing to offer loans when competing fiercely with others, even for lower credit-rating borrowers. Besides, as banks operating in highly concentrated systems reach more opportunities to earn better profits, they may lose incentives to try hard in their profit maximization strategies. Instead, banks may pursue safety and soundness objectives, which could be seen in the form of reduced risky investments, particularly fewer loans granted [27].

So far, some studies have explored the relationship between bank competition and the availability of bank credit, and mixed findings have been drawn. For example, some authors suggest that more powerful banks would probably cut their loan volume less than others in Eurozone countries [12, 13]. In contrast, some research reveals that a greater level of bank competition is linked with higher loan growth rates though researchers also look into the European banking [10]. It should be noted that such evidence is limited in emerging markets, where banking systems are different from those of developed economies.

Taken together, we respond to the mixed literature on the impact of bank competition on credit quantity by proposing the following hypotheses:

Hypothesis 2A. Higher competition increases the volume of bank credit.
Hypothesis 2B. Higher competition decreases the volume of bank credit.

### Bank competition and credit quality

The linkage between competition and bank risk has attracted much attention in the literature. According to the competition-fragility paradigm, previous studies highlight the adverse effects of increased competition [6]. In high-competitive markets, the pressure of banking competition causes a decrease in interest revenue and subsequently erodes bank profits. To maintain the return target set up by shareholders, bank managers are incentivized to take on more risk, ultimately hurting the quality of their loan portfolios. Moreover, as mentioned earlier, greater market power is ideal for banks to establish long-term relationships with borrowers. Such relationships could yield reduced information asymmetries through more accurate and detailed information about borrowers. This mechanism supports banks’ screening and monitoring activities, thereby improving their credit portfolios [24].

Another common mechanism in the literature to explain the competition-risk nexus is that dominant banks, in concentrated markets and under less competition pressure, may charge higher interest rates on loans, making it more challenging for their borrowers to repay the loans. This competition-stability paradigm may exacerbate the moral hazard problem and predict a reduction in the quality of banks’ asset portfolios [16]. Additionally, under the hypothesis of Mishkin [28], the “too-big-to-fail” philosophy is more pronounced in concentrated banking systems where dominant banks may involve in more risk-taking behaviors due to the assurance of government protection. This may cause more credit risk in a concentrated banking system.

In the context that predictions for the link between competition and credit risk are ambiguous theoretically, evidence found in empirical analyses also does not reach a consensus. On the one hand, previous papers document that higher bank competition may result in higher credit risk [14, 15]. Interestingly, this result is obtained for developing economies and transition countries that share a similar context with Vietnam. On the other hand, increased competition is demonstrated to improve asset quality in the US [29].

According to the above studies, competition can escalate or mitigate credit risks, leading to our final hypotheses as follows:

Hypothesis 3A. Higher competition increases the risk of bank credit.
Hypothesis 3B. Higher competition decreases the risk of bank credit.

## Data and methodology

### Data

Our primary bank-level data source for analysis is from the annual financial statements of Vietnamese commercial banks. A 15-year period from 2007 to 2021 is chosen given the data availability and considerable banking reforms, especially covering those related to World Trade Organization (WTO) participation. Apart from bank-level financial information, we collect macroeconomic data sources from the World Development Indicators (WDI) and the SBV. Given the small number of observations, we opt for the winsorization of the whole sample at the tails of 2.5th and 97.5th percentiles to deal with extreme outliers. As a result, we generate a sample with a total of 439 observations from 30 banks, forming an unbalanced panel dataset and making up over 90% of the banking system’s total assets in Vietnam in any given year during the 2007–2021 period under research.

### Bank competition measures

Various attempts have been made to quantify the banking sector’s structural characteristics [17, 18]. First, despite being widely used in empirical research, the structural indicators representing banking concentration exhibit major theoretical and practical flaws. Second, despite the fact that the H-statistic index could accurately measure competition from a static viewpoint, it also receives criticism for requiring assumptions that cannot be justified. Third, while the Boone index may emphasize the dynamic aspect of competition, it cannot characterize short-term competition. Lastly, though the Lerner index is good at highlighting individual banks’ market power, as opposed to other industry-level variables, it still has shortcomings and is not the most reliable competition metric. Furthermore, inferences regarding the level of competition based on various measures may vary substantially. The structural variables, for instance, concentrate on market concentration, the Lerner index regulates pricing power, and the H-statistic and Boone exploit the static and dynamic character of competition. Collectively, we contend that relying on a single bank competition measurement could be misleading. Hence, it is more appropriate to consider various alternative measures to comprehensively approach competition.

To capture bank competition, we build a series of different structural and non-structural measures. The structural approach can be further categorized into two sub-measures: the proportion of total banking sector assets from five leading banks (*CR5*) and the sum of each bank’s squared market shares in total banking market assets (*HHI*). In this study, we accept the prevalent theoretical proposition and suggest that a higher level of market concentration signals a lower degree of bank competition caused by greater market power by banks.

For the literature strand on bank competition in general and the non-structural approach in particular, the Lerner index could be seen as the most popular competition measure. Notably, it also captures banks’ market power and is a reverse proxy of bank competition. In a highly competitive market, a bank may have a product price equal to its marginal cost, thus possessing no market power. The Lerner index is generated from the function as follows:

$$\;Lerner{\;}_{it}\;=\;\frac{P_{it}\;-\;MC_{it}}{P_{it}}$$ (1)

Accordingly, different from the output price *P*_*it*_ (total revenues/total assets), marginal costs *MC*_*it*_ are purely estimated empirically from the following cost function, which contains one output (total assets) and three inputs (labor, funds, and physical capital):

$$lnCost_{it}\;=\;\beta_{0}\;+\;\beta_{1}\;lnQ_{it}\;+\;\frac{\beta_{2}}{2}\;lnQ_{it}^{2}\;+\;\sum_{k=1}^{3}\gamma_{it}\;lnW_{k,it}\;+\;\sum_{k=1}^{3}\delta_{k}\;lnQ_{it}\;lnW_{k,it}\;+\;\sum_{k=1}^{3}\sum_{j=1}^{3}lnW_{k,it}\;lnW_{j,it}\;+\;v_{t}\;+\;\varepsilon_{i,t}$$ (2)

Required coefficients are extracted from the estimation of the cost function to calculate the marginal costs using the formula:

$$MC_{it}\;=\;\frac{Cost_{it}}{Q_{it}}\;\left[\beta_{1}\;+\;\beta_{2}\;lnQ_{it}\;+\;\sum_{k=1}^{3}\delta_{k}\;lnW_{k,it}\right]$$ (3)

The most significant advantage of the Lerner index is that it is a bank-specific measure and varies over the years, thereby emphasizing the comparison of market power among banks over time. For a detailed method of calculating variables and estimating the cost function to reach the final Lerner index, please refer to previous papers [30–32].

Next, we employ micro-level data to estimate another non-structural competition indicator that captures the magnitude of the market share reallocation at the aggregate banking sector level, consistent with the theoretical innovation of Boone [33]. The empirical model in this vein is defined as follows:

$$\pi_{i}\;=\;\alpha \;+\;\beta \;ln\left(c_{i}\right)\;+\;\varepsilon_{i}$$ (4)

Where *π*_*i*_ and *c*_*i*_ are the profits and marginal costs of bank *i* (as a share of total assets), and *ε*_*i*_ is the error term. According to previous empirical constructions [18, 33], we gauge marginal costs by the ratio of average costs. With this setting, profits are larger at banks that own lower marginal costs (*β* < 0). We focus on *β* in absolute value to reveal the degree of competition; given that *β* typically receives negative values, an increase in the *β* estimate implies a decrease in the level of bank competition. We closely follow Schaeck and Cihák [34] to apply the system generalized method of moments (GMM) to regress our Boone model.

Finally, we opt for the H-statistic approach proposed by Panzar and Rosse [35] to gauge bank competition. Under this non-structural approach, the H-statistic indicator is gained by estimating the function:

$$lnP_{it}\;=\;\beta_{0}\;+\;\beta_{1}\;lnW_{1,it}\;+\;\beta_{2}\;lnW_{2,it}\;+\;\beta_{3}\;lnW_{3,it}\;+\;\gamma_{1}\;lnY_{1,it}+\gamma_{2}\;lnY_{2,it}\;+\;\gamma_{3}\;lnY_{3,it}\;+\;\delta D\;+\;\varepsilon_{i,t}$$ (5)

The dependent variable *P*_*it*_ is the share of gross interest income to total assets. Three main input prices consist of funding costs, personnel costs, and physical capital costs. We also follow the standard practice to allow some bank-specific controls in the model, including the ratio of loans to total assets (*Y*_*1*,*it*_), the ratio of equity to total assets (*Y*_*2*,*it*_), and total assets (*Y*_*3*,*it*_). All variables are in the logarithm form. The year dummy variable is also added (*D*). The level of bank competition according to this H-statistic approach is specified by the sum of *β*_*1*_, *β*_*2*_, and *β*_*3*_. Our final variable is obtained from pooled ordinary least squares (OLS) estimation with time dummies (*H1*), and we also allow for the generalized least squares (GLS) regression to generate an additional proxy of bank competition (*H2*), based on the Panzar-Rosse H-statistic index [36]. To justify the use of the Panzar-Rosse H-statistic index, our test needs to ensure that the market is in long-run equilibrium [36]. Similar to Guidi [37], our checks for a long-run equilibrium indicate that this condition is verified in the banking industry under study.

### Model specification and econometric technique

To answer our research questions, we regress our dependent variables (*Y*) for different bank lending dimensions on separate measures identifying the banking market structure (*COMP*), while controlling for key factors at both micro- and macro-level (*X* and *Z*) as follows:

$$Y_{i,t}\;=\;\alpha_{0}\;+\;\alpha_{1}\;\times \;Y_{i,t-1}\;+\;\alpha_{2}\;\times \;COMP_{t-1}\;+\;\alpha_{3}\;\times \;X_{i,t-1}\;+\;\alpha_{4}\;\times \;Z_{t-1}\;+\;v_{i}\;+\;\varepsilon_{i,t}$$ (6)

where *i* and *t* refer to banks and years, respectively. Dependent variables capture the aspects of credit volume (the annual change in gross loans), credit risk (the ratio of loan loss provisions to gross loans), and credit price (the ratio of total interest income to gross loans). These are straightforward measures and have been widely employed in the banking literature to measure lending activities. We include numerous bank-level and country-level control variables in line with the relevant literature [38–40]. As bank-level control variables, we consider bank liquidity (share of liquid assets in total assets), bank size (natural logarithm of total bank assets), capital (share of equity in total assets), and diversification (share of non-interest income in operating income). For country-level variables, we allow for economic cycles (the annual real GDP growth rate), monetary policy (refinancing rates announced by the central bank), and financial crisis (dummy variable taking a value of 1 for observation years in 2007–2009 and zero otherwise).*v*_*i*_ indicates bank fixed effects, and *ε*_*i*,*t*_ is an error term. We do not insert a set of the time-fixed effects as we alternatively include economic cycles and crisis dummies, gauging bank-invariant effects specific to each observation year.

The inclusion of the lagged dependent variable (*Y*_*i*,*t-1*_) in our model exhibits the dynamic nature of bank lending activities, and we have to prefer the choice of the two-step system GMM when conducting estimations to handle the issue of endogeneity [41]. This approach with an instrumental variable technique helps to avoid biased and inconsistent estimates, which fail to be obtained if regressed by traditional fixed or random effects. We have to ensure the consistency of our dynamic GMM by the Arellano-Bond tests, required to justify the absence of the second-order autocorrelation, and by the Hansen test, demanded to verify the joint validity of instruments. For the estimation technique, we use the “xtabond2” STATA command with small sample and instrument collapse options to limit the number of instruments [42].

### Preliminary statistics

Table 1 presents the variables’ summary distributions. The Vietnamese banking system shows a high degree of concentration, with only a few large banks constituting most of the sector’s total assets, as depicted by a mean of 0.586 of the five-bank concentration ratio (*CR5*). However, the picture appears to be different if we rely on other indicators that gauge banking market structure. Specifically, different from the *CR5* variable, the *HHI* market concentration index offers evidence of an un-concentrated market based on an average of 0.088. Other measures (the Lerner index with a mean of 0.349, the Boone index with a mean of -0.079, and the H-statistic indicator (*H1*) with a mean of 0.491) help to exhibit a moderate competitive nature of the banking market. These preliminary reports are plausible with our identification strategy of not relying on one single competition measure, as each of the different measures focuses on one particular aspect of the banking market structure. Besides, our general market statistics of Vietnam are different from those of the European banking sectors shown in previous studies [17, 22] but share some similarities with several works on emerging markets [39, 43].

**Table 1:** Descriptive statistics.

	Mean	SD	Min	Max	
Loan growth rate	27.579	26.518	-2.358	101.758	The annual change in gross loans
Loan loss provisions	0.959	0.672	0.122	2.516	The ratio of loan loss provisions to gross loans
Interest to loans	14.235	5.409	8.541	28.107	The ratio of total interest income to gross loans
Lerner	0.349	0.089	0.195	0.518	Bank market power by relating price to marginal cost
CR5	0.586	0.052	0.539	0.713	The proportion of total banking sector assets from five leading banks
HHI	0.088	0.015	0.075	0.125	The sum of each bank’s squared market shares in total banking market assets
Boone	-0.079	0.003	-0.086	-0.073	Boone competition indicator, based on profit efficiency in the banking sector
H1	0.491	0.118	0.372	0.735	H-statistics competition indicator, obtained from pooled OLS estimation
H2	0.575	0.085	0.472	0.745	H-statistics competition indicator, obtained from fixed effects GLS estimation
Liquidity	33.562	10.791	17.719	56.053	The share of liquid assets in total assets
Size	32.144	1.262	29.961	34.486	The natural logarithm of total bank assets
Capital	9.696	4.293	4.859	20.470	The share of equity in total assets
Diversification	0.239	0.143	0.002	0.575	The share of non-interest income in operating income
GDP	5.795	1.323	2.580	7.130	The annual real GDP growth rate
Policy rates	7.513	2.734	4.000	15.000	Refinancing rates announced by the central bank
Crisis dummy	0.189	0.392	0.000	1.000	Dummy variable taking a value of 1 for observation years in 2007–2009 and zero otherwise

## Empirical results

Subsequent subsections exhibit the regression results of different lending functions. Overall, all estimations of the dynamic GMM reveal strong evidence of the instrument’s validity and no second-order autocorrelation. Besides, the highly significant coefficient of lagged lending measures is confirmed in all results. Hence, we gain enough confidence in the consistency of the dynamic model specification estimated by the system GMM technique.

### The effects of bank competition on credit prices

The regression results in Table 2 show the relationship between different measures of competition and credit prices. We first observe the significant positive coefficient on the Lerner index. This result implies that greater market power increases the ratio of interest to loans, confirming that increased competition may reduce banks’ credit prices.

**Table 2:** The effects of bank competition on credit price.

	Dependent variable: Interest to loans					
	(1)	(2)	(3)	(4)	(5)	(6)
Lagged dependent variable	0.251***	1.626***	0.156**	0.081***	0.056***	0.038***
	(0.063)	(0.580)	(0.077)	(0.017)	(0.013)	(0.014)
Lerner	37.795***					
	(5.734)					
CR5		-44.217***				
		(6.321)				
HHI			-116.552***			
			(22.915)			
Boone				-360.763		
				(267.477)		
H1					24.089***	
					(4.249)	
H2						15.295***
						(4.199)
Liquidity	0.096***	0.374***	0.339***	0.434***	0.412***	0.396***
	(0.029)	(0.030)	(0.035)	(0.033)	(0.036)	(0.038)
Size	-7.181***	-4.262***	-4.566***	-3.684***	-3.805***	-3.947***
	(0.636)	(0.625)	(0.694)	(0.558)	(0.593)	(0.573)
Capital	-1.215***	-0.739***	-0.738***	-0.599***	-0.740***	-0.665***
	(0.163)	(0.130)	(0.137)	(0.126)	(0.127)	(0.124)
Diversification	11.610***	10.793***	12.446***	7.429***	6.010***	6.759***
	(1.691)	(1.674)	(1.547)	(1.961)	(2.056)	(1.958)
GDP	0.031	0.665***	0.483***	0.164**	0.292***	0.267***
	(0.054)	(0.057)	(0.062)	(0.064)	(0.071)	(0.069)
Policy rates	0.461***	0.251**	0.282**	0.261**	0.142	0.087
	(0.091)	(0.122)	(0.119)	(0.112)	(0.113)	(0.099)
Crisis dummy	-6.613***	-1.407*	-0.025	-4.501***	-5.906***	-4.646***
	(0.782)	(0.742)	(0.636)	(0.598)	(0.723)	(0.614)
Observations	409	409	409	409	409	409
Banks	30	30	30	30	30	30
Instruments	28	28	28	28	28	28
AR(1) test	0.000	0.000	0.002	0.004	0.002	0.002
AR(2) test	0.201	0.416	0.151	0.328	0.317	0.280
Hansen test	0.111	0.138	0.139	0.201	0.144	0.209

Note: The sample data run from 2007 to 2021. All regressions are obtained from the system GMM estimator. Robust standard errors are shown in brackets. Diagnostic tests are reports with p-values. (***, **, *) establish the significance levels at 1%, 5%, and 10%.

However, when employing the *CR5* and *HHI* ratios as measures of concentration, we document that increasing market concentration leads to a decrease in the ratio of interest to loans, shown by the significant negative coefficients on the *CR5* and *HHI* variables; alternatively speaking, higher competition may increase the price of credit. This finding is repeated when we use two alternative variables based on the H-statistic index to capture bank competition. Concretely, the coefficients on both *H1* and *H2* variables are significantly positive, suggesting that higher competition may raise credit costs. Different from all measures discussed above, the Boone indicator does not have a statistically significant influence on the cost of credit.

Overall, our estimates ascertain that different measures of banking market structure have different impacts on credit prices. The results with the structural proxies, *HHI* and *CR5*, move in the same direction and signal the positive link between competition and credit price. For the non-structural proxies, while the Lerner index and the H-statistic indicator offer contrasting results, the Boone index is not a significant factor in explaining the cost of credit. These results indicate no clear effect of competition on bank credit prices in Vietnam. Our finding for the link between market structure and credit prices cannot lend support to the theory on the benefits of competition, which states that the more competitive the market, the lower the price. In this case, higher competition in the banking sector does not necessarily cause bank prices to decrease. This may be due to the fact that Vietnamese banks operate under the strict control of the SBV, especially in the interest rate framework, which makes banks with different profiles of market power and lending capacity face the same difficulty in adjusting interest rates flexibly. Our mixed estimates on the impact of multiple competition measures are in line with some previous studies [18, 30, 44, 45], thereby supporting the view that the conclusion could be misleading if we only rely on one competition measure.

### The effects of bank competition on credit quantity

Table 3 exhibits our estimation results for the effects of bank competition on credit quantity. Bank market power, as reflected by the Lerner index, has a positive and statistically significant coefficient in column 1. This result implies that higher market power boosts the loan growth rate of banks, thus supporting the negative link between competition and credit volume since the Lerner index is a reverse proxy of bank competition. As shown in columns 2–3, the coefficients on market concentration variables–the Herfindahl-Hirschman indicator and the 5-bank concentration index, are positive and significant. These estimates reveal that banks in more concentrated markets expand their loans to a larger extent. As a common practice, concentration ratios can serve as structural competition measurements. Under this structural approach, we could infer that greater competition is associated with less loan growth.

**Table 3:** The effects of bank competition on credit quantity.

	Dependent variable: Loan growth rate					
	(1)	(2)	(3)	(4)	(5)	(6)
Lagged dependent variable	-0.073***	-0.130***	-0.146***	-0.125***	-0.207***	-0.240***
	(0.021)	(0.022)	(0.023)	(0.018)	(0.034)	(0.033)
Lerner	75.281***					
	(15.939)					
CR5		67.368**				
		(33.171)				
HHI			407.793***			
			(120.545)			
Boone				1,868.651**		
				(738.051)		
H1					-172.399***	
					(12.678)	
H2						-169.236***
						(12.332)
Liquidity	-0.359***	-0.197	-0.230	-0.220*	-0.243	-0.168
	(0.126)	(0.133)	(0.140)	(0.116)	(0.175)	(0.148)
Size	-12.408***	-5.830**	-6.426***	-7.302***	-6.270	-2.064
	(1.909)	(2.370)	(2.248)	(2.018)	(4.343)	(4.264)
Capital	3.114***	1.898***	2.276***	1.620***	4.561***	4.477***
	(0.342)	(0.563)	(0.605)	(0.599)	(1.046)	(1.000)
Diversification	-25.337***	-39.019***	-46.551***	-32.159***	-4.196	-1.270
	(6.827)	(12.247)	(12.601)	(9.379)	(12.913)	(12.250)
GDP	-0.453	-1.051***	-0.720**	-1.866***	-2.941***	-2.997***
	(0.360)	(0.390)	(0.364)	(0.227)	(0.492)	(0.486)
Policy rates	0.579**	0.305	0.154	0.432	6.202***	5.683***
	(0.245)	(0.228)	(0.259)	(0.266)	(0.485)	(0.471)
Crisis dummy	30.340***	29.455***	33.933***	19.689***	21.391***	18.101***
	(1.774)	(6.834)	(6.124)	(4.089)	(5.315)	(5.293)
Observations	401	401	401	401	401	401
Banks	30	30	30	30	30	30
Instruments	28	28	28	28	28	28
AR(1) test	0.001	0.001	0.000	0.001	0.001	0.001
AR(2) test	0.187	0.240	0.182	0.254	0.304	0.362
Hansen test	0.121	0.196	0.170	0.226	0.202	0.189

Note: The sample data run from 2007 to 2021. All regressions are obtained from the system GMM estimator. Robust standard errors are shown in brackets. Diagnostic tests are reports with p-values. (***, **, *) establish the significance levels at 1%, 5%, and 10%.

In the other regressions, we find a significantly positive coefficient on the Boone index and a significantly negative coefficient on the H-statistic variables. Given that an increase in the H-statistic index suggests greater competition and the Boone index is a reverse proxy of bank competition, our set of results in this case indicates higher banking competition results in less credit granted.

Taken together, all of the regression results analyzed above highlight a consistent relationship where bank competition is found to dampen the expansion of bank loans. This finding confirms Hypothesis 2B. Potentially, we can rely on the mechanism of close customer relationships and better funding access at banks with higher market power to justify a negative link between bank competition and average loan growth [24, 26]. Moreover, excessive competition can make Vietnamese banks more vulnerable, particularly weak ones, making the banking market more cautious in lending activities, which are closely related to potential risk. The estimates are economically valid as well. In more detail, for instance, an increase of one standard deviation in market power (column 1), all other factors being equal, is associated with a rise of 6.7 percentage points in the loan growth rate (i.e., the coefficient of 75.281 multiplied by the standard deviation of the Lerner index, 0.089). Taking another example based on column 2, with a one standard deviation increase in the concentration ratio (measured as *CR5*), the loan growth rate falls by 3.503 percentage points (67.368*0.052).

### The effects of bank competition on credit quality

Table 4 presents the regression results for the model of credit risk. According to columns 1–4, different banking market structure variables, including the Lerner index, concentration measures, and the Boone indicator, are all negatively linked with loan loss provisions–a measure of credit risk. When all of these market structure variables serve well as reverse proxies of bank competition, and the coefficients are all statistically significant, we could suggest that a greater level of competition in the market may increase credit risk.

**Table 4:** The effects of bank competition on credit quality.

	Dependent variable: Loan loss provisions					
	(1)	(2)	(3)	(4)	(5)	(6)
Lagged dependent variable	0.343***	0.409***	0.423***	0.326***	0.404***	0.373***
	(0.055)	(0.071)	(0.068)	(0.058)	(0.059)	(0.058)
Lerner	-1.146***					
	(0.263)					
CR5		-1.691***				
		(0.622)				
HHI			-5.490**			
			(2.213)			
Boone				-35.417***		
				(12.010)		
H1					1.078***	
					(0.292)	
H2						1.082***
						(0.277)
Liquidity	0.012***	0.005	0.005	0.005	0.001	0.003
	(0.004)	(0.005)	(0.005)	(0.004)	(0.005)	(0.005)
Size	-0.184***	-0.165***	-0.173***	-0.187***	-0.194***	-0.219***
	(0.042)	(0.050)	(0.050)	(0.041)	(0.038)	(0.034)
Capital	-0.077***	-0.079***	-0.076***	-0.076***	-0.087***	-0.087***
	(0.016)	(0.007)	(0.008)	(0.008)	(0.008)	(0.008)
Diversification	1.217***	1.454***	1.461***	1.529***	1.479***	1.588***
	(0.273)	(0.352)	(0.341)	(0.296)	(0.317)	(0.296)
GDP	-0.098***	-0.076***	-0.079***	-0.097***	-0.080***	-0.078***
	(0.012)	(0.015)	(0.015)	(0.012)	(0.013)	(0.013)
Policy rates	0.043***	0.039***	0.038***	0.045***	0.004	0.008
	(0.005)	(0.009)	(0.009)	(0.008)	(0.014)	(0.014)
Crisis dummy	-0.223***	0.086	0.061	-0.184***	-0.194***	-0.180***
	(0.052)	(0.063)	(0.060)	(0.057)	(0.052)	(0.051)
Observations	409	409	409	409	409	409
Banks	30	30	30	30	30	30
Instruments	28	28	28	28	28	28
AR(1) test	0.004	0.002	0.002	0.001	0.003	0.000
AR(2) test	0.214	0.266	0.188	0.308	0.370	0.364
Hansen test	0.224	0.176	0.193	0.187	0.170	0.182

Note: The sample data run from 2007 to 2021. All regressions are obtained from the system GMM estimator. Robust standard errors are shown in brackets. Diagnostic tests are reports with p-values. (***, **) establish the significance levels at 1% and 5%.

For the remaining estimations in the equation of the provisions, we find that the coefficients on the H-statistic variants are positive and statistically significant. As this methodology clarifies that an increase in the H-statistic index highlights higher competition, our result shows that there could be a positive relationship between bank competition and credit risk.

In sum, the estimation results from various competition measures presented in Table 4 consistently reveal that greater competition may cause the banking sector to suffer a higher level of credit risk or a deterioration in credit quality. The magnitude of our coefficients indicates that the impacts documented are economically sizable. For instance, looking at the coefficient in column 4, we could estimate that a one-standard-deviation decrease in the Boone index (increased competition) may cause an increase in the provision ratio by 0.106 percentage points (35.417*0.003). Similarly, in response to a one standard deviation increase in the competition level by the H-statistic variable (column 5, *H1*), banks’ credit risk may increase by 0.127 percentage points (1.078*0.118). Our finding is in line with the competition-fragility paradigm, supporting Hypothesis 3A. The enormous pressure of competition may make banks fail to select, screen, and monitor a riskier pool of borrowers [6]. This can be an essential explanation in line with the Vietnamese banking practice, where competitive pressures have forced banks to loosen lending policies and thereby lead to declines in loan quality.

### Robustness checks

Our estimates thus far show that increased competition in the banking market not only reduces the volume of bank credit but also increases the level of credit risk, in the context that no evidence of the change in credit price is obtained. We further confirm such adverse effects of bank competition using many additional tests in this part. With all robustness checks provided in Tables 5–9, we only display the results for variables of primary interest to save space, though all regressions are re-estimated with full regressors.

**Table 5:** Robustness tests using alternative bank competition and lending price measures.

	Dependent variable: Interest to loans							
	(1)	(2)	(3)	(4)	(5)	(6)	(7)	(8)
	Lerner_adjusted	CR5_deposit	CR3_deposit	HHI_deposit	Boone1	Boone2	H3	H4
Lagged dependent variable	0.384***	1.016***	1.020***	0.108**	0.064***	0.067***	0.281***	0.284***
	(0.077)	(0.365)	(0.392)	(0.047)	(0.014)	(0.014)	(0.091)	(0.086)
Bank competition	27.666***	-66.816***	-61.698***	-187.701***	-425.340	-514.291*	43.163***	43.526***
	(5.773)	(6.318)	(7.786)	(26.722)	(263.004)	(308.077)	(3.455)	(3.672)
Bank controls	Yes	Yes	Yes	Yes	Yes	Yes	Yes	Yes
Macro controls	Yes	Yes	Yes	Yes	Yes	Yes	Yes	Yes
Observations	409	409	409	409	409	409	409	409
Banks	30	30	30	30	30	30	30	30
Instruments	28	28	28	28	28	28	28	28
AR(1) test	0.001	0.001	0.001	0.001	0.003	0.003	0.001	0.000
AR(2) test	0.149	0.131	0.353	0.171	0.197	0.125	0.425	0.156
Hansen test	0.122	0.128	0.165	0.117	0.221	0.148	0.205	0.137
	Dependent variable: NIM							
	(9)	(10)	(11)	(12)	(13)	(14)		
	Lerner	CR5	HHI	Boone	H1	H2		
Lagged dependent variable	0.470***	1.152***	1.124***	1.011***	1.011***	0.975***		
	(0.087)	(0.132)	(0.131)	(0.124)	(0.122)	(0.121)		
Bank competition	4.731***	-4.010***	-8.931**	-14.947	1.527***	1.150*		
	(1.048)	(1.382)	(3.970)	(28.075)	(0.515)	(0.588)		
Bank controls	Yes	Yes	Yes	Yes	Yes	Yes		
Macro controls	Yes	Yes	Yes	Yes	Yes	Yes		
Observations	409	409	409	409	409	409		
Banks	30	30	30	30	30	30		
Instruments	28	28	28	28	28	28		
AR(1) test	0.003	0.004	0.002	0.000	0.002	0.003		
AR(2) test	0.253	0.382	0.275	0.384	0.297	0.296		
Hansen test	0.183	0.114	0.115	0.142	0.182	0.224		

Note: The sample data run from 2007 to 2021. All regressions are obtained from the system GMM estimator. Robust standard errors are shown in brackets. Diagnostic tests are reports with p-values. (***, **, *) establish the significance levels at 1%, 5%, and 10%.

**Table 6:** Robustness tests using alternative bank competition and lending volume measures.

	Dependent variable: Loan growth rate							
	(1)	(2)	(3)	(4)	(5)	(6)	(7)	(8)
	Lerner_adjusted	CR5_deposit	CR3_deposit	HHI_deposit	Boone1	Boone2	H3	H4
Lagged dependent variable	-0.116***	-0.110***	-0.133***	-0.136***	-0.127***	-0.131***	-0.182***	-0.232***
	(0.030)	(0.033)	(0.032)	(0.032)	(0.018)	(0.017)	(0.028)	(0.032)
Bank competition	63.609***	342.337***	357.075***	1,075.513***	1,870.329***	2,145.358***	-205.257***	-225.067***
	(6.164)	(20.033)	(24.689)	(79.617)	(717.033)	(721.685)	(10.162)	(13.528)
*Bank controls*	*Yes*	*Yes*	*Yes*	*Yes*	*Yes*	*Yes*	*Yes*	*Yes*
*Macro controls*	*Yes*	*Yes*	*Yes*	*Yes*	*Yes*	*Yes*	*Yes*	*Yes*
Observations	401	401	401	401	401	401	401	401
Banks	30	30	30	30	30	30	30	30
Instruments	28	28	28	28	28	28	29	30
AR(1) test	0.000	0.000	0.000	0.000	0.000	0.000	0.000	0.000
AR(2) test	0.123	0.250	0.228	0.332	0.326	0.341	0.331	0.214
Hansen test	0.191	0.145	0.204	0.151	0.145	0.190	0.143	0.124
	Dependent variable: Loan share							
	(9)	(10)	(11)	(12)	(13)	(14)		
	Lerner	CR5	HHI	Boone	H1	H2		
Lagged dependent variable	0.946***	0.535***	0.630***	0.652***	0.675***	0.672***		
	(0.098)	(0.108)	(0.104)	(0.105)	(0.109)	(0.115)		
Bank competition	17.014***	83.676***	272.488***	305.980**	-11.141***	-12.191***		
	(4.097)	(13.373)	(48.520)	(133.248)	(4.161)	(4.076)		
*Bank controls*	*Yes*	*Yes*	*Yes*	*Yes*	*Yes*	*Yes*		
*Macro controls*	*Yes*	*Yes*	*Yes*	*Yes*	*Yes*	*Yes*		
Observations	409	409	409	409	409	409		
Banks	30	30	30	30	30	30		
Instruments	28	28	28	28	28	28		
AR(1) test	0.002	0.002	0.001	0.003	0.003	0.001		
AR(2) test	0.122	0.359	0.191	0.188	0.263	0.145		
Hansen test	0.222	0.175	0.140	0.197	0.144	0.218		

Note: The sample data run from 2007 to 2021. All regressions are obtained from the system GMM estimator. Robust standard errors are shown in brackets. Diagnostic tests are reports with p-values. (***, **) establish the significance levels at 1% and 5%.

**Table 7:** Robustness tests using alternative bank competition and lending quality measures.

	Dependent variable: Loan loss provisions							
	(1)	(2)	(3)	(4)	(5)	(6)	(7)	(8)
	Lerner_adjusted	CR5_loan	CR3_loan	HHI_loan	Boone1	Boone2	H3	H4
Lagged dependent variable	0.458***	0.613***	0.541***	0.528***	0.316***	0.314***	0.486***	0.479***
	(0.060)	(0.043)	(0.054)	(0.054)	(0.059)	(0.058)	(0.057)	(0.060)
Bank competition	-0.647***	-3.718***	-2.981***	-8.433***	-43.809***	-56.258***	1.325***	1.401***
	(0.241)	(0.445)	(0.669)	(1.920)	(12.290)	(14.304)	(0.238)	(0.286)
*Bank controls*	*Yes*	*Yes*	*Yes*	*Yes*	*Yes*	*Yes*	*Yes*	*Yes*
*Macro controls*	*Yes*	*Yes*	*Yes*	*Yes*	*Yes*	*Yes*	*Yes*	*Yes*
Observations	409	409	409	409	409	409	409	409
Banks	30	30	30	30	30	30	30	30
Instruments	28	28	28	28	28	28	28	28
AR(1) test	0.003	0.000	0.003	0.000	0.002	0.001	0.002	0.000
AR(2) test	0.383	0.353	0.196	0.306	0.346	0.356	0.309	0.392
Hansen test	0.133	0.174	0.157	0.177	0.125	0.219	0.156	0.175
	Dependent variable: Non-performing loans							
	(9)	(10)	(11)	(12)	(13)	(14)		
	Lerner	CR5	HHI	Boone	H1	H2		
Lagged dependent variable	0.361***	0.506***	0.383***	0.457***	0.315***	0.279***		
	(0.062)	(0.076)	(0.058)	(0.077)	(0.040)	(0.035)		
Bank competition	-5.065**	-2.383*	-19.430***	-68.025***	2.688***	2.044**		
	(2.345)	(1.395)	(4.952)	(25.887)	(0.892)	(0.883)		
*Bank controls*	*Yes*	*Yes*	*Yes*	*Yes*	*Yes*	*Yes*		
*Macro controls*	*Yes*	*Yes*	*Yes*	*Yes*	*Yes*	*Yes*		
Observations	355	355	355	355	355	355		
Banks	30	30	30	30	30	30		
Instruments	28	28	28	28	28	28		
AR(1) test	0.004	0.003	0.002	0.003	0.002	0.004		
AR(2) test	0.183	0.248	0.291	0.377	0.196	0.380		
Hansen test	0.147	0.143	0.165	0.167	0.136	0.231		

Note: The sample data run from 2007 to 2021. All regressions are obtained from the system GMM estimator. Robust standard errors are shown in brackets. Diagnostic tests are reports with p-values. (***, **, *) establish the significance levels at 1%, 5%, and 10%.

**Table 8:** Subsample tests.

	Dependent variable: Loan growth rate						Dependent variable: Loan loss provisions					
	(1)	(2)	(3)	(4)	(5)	(6)	(7)	(8)	(9)	(10)	(11)	(12)
	Lerner	CR5	HHI	Boone	H1	H2	Lerner	CR5	HHI	Boone	H1	H2
Lagged dependent variable	-0.082***	-0.078***	-0.143**	-0.134**	-0.132**	-0.174***	0.513***	0.490***	0.508***	0.572***	0.532***	0.547***
	(0.029)	(0.014)	(0.057)	(0.058)	(0.063)	(0.063)	(0.028)	(0.074)	(0.075)	(0.057)	(0.052)	(0.059)
Bank competition	94.235***	104.235***	730.292***	7,039.703***	-156.313***	-172.085***	-0.465**	-1.287*	-3.087	-31.276***	1.144**	0.841**
	(13.062)	(25.132)	(116.766)	(1,208.060)	(48.554)	(47.931)	(0.200)	(0.681)	(2.015)	(11.458)	(0.502)	(0.353)
*Bank controls*	*Yes*	*Yes*	*Yes*	*Yes*	*Yes*	*Yes*	*Yes*	*Yes*	*Yes*	*Yes*	*Yes*	*Yes*
*Macro controls*	*Yes*	*Yes*	*Yes*	*Yes*	*Yes*	*Yes*	*Yes*	*Yes*	*Yes*	*Yes*	*Yes*	*Yes*
Observations	296	296	296	296	296	296	298	298	298	298	298	298
Banks	30	30	30	30	30	30	30	30	30	30	30	30
Instruments	22	22	22	22	22	22	22	22	22	22	22	22
AR(1) test	0.000	0.001	0.004	0.002	0.002	0.004	0.001	0.003	0.002	0.002	0.000	0.001
AR(2) test	0.356	0.416	0.125	0.377	0.154	0.193	0.278	0.290	0.377	0.337	0.326	0.336
Hansen test	0.190	0.189	0.159	0.110	0.210	0.154	0.132	0.189	0.172	0.168	0.163	0.169
	Dependent variable: Interest to loans											
	(13)	(14)	(15)	(16)	(17)	(18)						
	Lerner	CR5	HHI	Boone	H1	H2						
Lagged dependent variable	0.474***	0.146**	0.286***	0.375***	0.526***	0.707***						
	(0.067)	(0.068)	(0.074)	(0.130)	(0.129)	(0.126)						
Bank competition	14.396**	-59.335***	-204.552***	490.813	19.917**	38.549***						
	(5.777)	(4.922)	(20.757)	(309.863)	(8.276)	(8.903)						
*Bank controls*	*Yes*	*Yes*	*Yes*	*Yes*	*Yes*	*Yes*						
*Macro controls*	*Yes*	*Yes*	*Yes*	*Yes*	*Yes*	*Yes*						
Observations	298	298	298	298	298	298						
Banks	30	30	30	30	30	30						
Instruments	22	22	22	22	22	22						
AR(1) test	0.004	0.004	0.004	0.000	0.001	0.004						
AR(2) test	0.378	0.175	0.406	0.233	0.280	0.335						
Hansen test	0.120	0.176	0.196	0.150	0.130	0.212						

Note: The sample data run from 2010 to 2019. All regressions are obtained from the system GMM estimator. Robust standard errors are shown in brackets. Diagnostic tests are reports with p-values. (***, **, *) establish the significance levels at 1%, 5%, and 10%.

**Table 9:** Dynamic LSDVC estimates.

	Dependent variable: Loan growth rate					Dependent variable: Loan loss provisions				
	(1)	(2)	(3)	(4)	(5)	(6)	(7)	(8)	(9)	(10)
	Lerner	CR5	HHI	H1	H2	Lerner	CR5	HHI	H1	H2
Lagged dependent variable	-0.080***	-0.088***	-0.103***	-0.172***	-0.204***	0.280***	0.317***	0.332***	0.300***	0.292***
	(0.020)	(0.024)	(0.022)	(0.032)	(0.029)	(0.055)	(0.084)	(0.083)	(0.085)	(0.086)
Bank competition	65.205***	172.078***	329.020***	-140.904***	-135.284***	-1.118***	-1.511***	-4.551***	0.498**	0.713***
	(21.671)	(8.971)	(109.753)	(10.395)	(9.003)	(0.218)	(0.360)	(1.182)	(0.245)	(0.275)
*Bank controls*	*Yes*	*Yes*	*Yes*	*Yes*	*Yes*	*Yes*	*Yes*	*Yes*	*Yes*	*Yes*
*Macro controls*	*Yes*	*Yes*	*Yes*	*Yes*	*Yes*	*Yes*	*Yes*	*Yes*	*Yes*	*Yes*
Observations	401	401	401	401	401	409	409	409	409	409
Banks	30	30	30	30	30	30	30	30	30	30
	Dependent variable: Interest to loans									
	(11)	(12)	(13)	(14)	(15)					
	Lerner	CR5	HHI	H1	H2					
Lagged dependent variable	0.195***	0.165**	0.177**	0.156***	0.144***					
	(0.065)	(0.071)	(0.073)	(0.043)	(0.044)					
Bank competition	32.492***	-39.921***	-104.417***	21.030***	13.542***					
	(6.239)	(5.246)	(20.037)	(3.377)	(3.233)					
*Bank controls*	*Yes*	*Yes*	*Yes*	*Yes*	*Yes*					
*Macro controls*	*Yes*	*Yes*	*Yes*	*Yes*	*Yes*					
Observations	409	409	409	409	409					
Banks	30	30	30	30	30					

Note: The sample data run from 2007 to 2021. All regressions are obtained from the dynamic LSDVC estimator. Bootstrapped standard errors are shown in brackets. Technically, the time-invariant Boone index is dropped under the LSDVC estimation. (***, **) establish the significance levels at 1% and 5%.

First, we alter our ways of measuring key variables of interest. We first deal with the criticism of the Lerner index construction, arguing that market power might be overestimated as banks’ pricing power in deposit markets is transferrable to lending markets [46]. To tackle this issue, we follow Turk Ariss [31], who proposes a simple way by wiping out the cost of funds from the translog cost function. Concerning concentration ratios, we look into the dimensions of deposits and loans while performing calculations instead of total assets, as applied earlier. Another variant from the perspective of the top three banks (*CR3*) is also studied along with the original *CR5* ratio. As for the alternative variables of the H-statistics index, we follow Claessens and Laeven [36] to replace the present dependent variable (gross interest income) with the total income, while we still keep the estimation technique to regress our new models. In this regard, we obtain two new alternative competition variables based on the spirit of the H-statistics index, denoted as *H3* (pooled OLS) and *H4* (fixed effects). Besides, with respect to the alternative measures based on the spirit of the Boone index, we utilize the strategy as follows: (i) we choose to obtain the marginal cost factor from the translog cost estimation rather than traditionally relying on the average costs; (ii) we use a new dependent variable by replacing bank profits with their market shares. The newly-established model is then estimated using the GMM style and the pooled OLS tool, constituting *Boone1* and *Boone2* proxies, respectively.

Turning to the adjusted measurement of banking lending variables, we alternatively represent the costs of credit by the net interest margin (net interest income divided by interest-earning assets), the access to credit by gross loans divided by total assets, and credit quality by non-performing loans divided by gross loans. We replace previous dependent variables with these newly-established ones in the regression models and repeat the estimation procedures as outlined earlier. Our results with alternative measures are reported in Tables 5–7, consistently highlighting robust patterns: we still observe that competition in the banking market is negatively associated with credit expansion and positively linked with credit risk, while the inconclusive finding on credit costs is unchanged. Some loan- and deposit-based concentration ratios are not presented to save space.

Second, we perform robustness tests in the subsample. This way of analysis is motivated by the fact that our data sample contains two substantial potential structural breaks, including the global financial crisis of 2007–2009 and the COVID-19 pandemic period of 2020–2021, which may significantly alter banks’ lending behaviors and market structures. To address this point, we adjust our sample data by eliminating these two periods of financial and health crises. Estimation results in the new subsample are presented in Table 8, showing that our obtained findings survive regardless of the likely structural breaks caused by the 2007–2009 financial crisis and the 2020–2021 COVID-19 pandemic.

Third, we change our econometric approach. Given some weaknesses of our dataset, i.e., the number of cross-sections is small and the panel is heavily unbalanced, one could underestimate the estimation power of the dynamic GMM technique, as demonstrated in multiple previous works [47–49]. This inspires us to use an alternative econometric technique to overcome the current issue. In this regard, we introduce the corrected least square dummy variable (LSDVC) estimator that works well with our sample data as it is featured by higher root mean square errors (RMSE) that can help to offer less biased and more efficient estimates [50]. Consistent with prior studies [50, 51], we establish the LSDVC coefficients by applying the bootstrapped standard errors with 50 iterations. For brevity, we only report the results with LSDVC coefficients estimated on the basis of the Blundell-Bond generation (while other LSDVC generations remain the same). Results are reported in Table 9, and once again we observe that the coefficients on the banking market structure variables are significant in most columns with unaltered signs.

### Heterogeneity analysis: Bank diversification

This paper introduces two key objectives: (i) to explore the linkage between market structure and bank lending aspects and (ii) to analyze whether this linkage is similar across different banks of different strategic business lines. Hence, after showing a decline in bank lending volume and an increase in credit risk under the environment of high competition, in this subsection we desire to pay attention to the moderating role of bank diversification. The important motivation behind this analysis is that diversification is beneficial to the reduction of information asymmetries and idiosyncratic shocks [52]. Furthermore, diversification is a great tool to increase the economies of scope and cross-selling activities [53]. In this regard, we can expect bank diversification to help banks tackle any challenge caused by high competition in the market. However, banks are also advised against diversification and instead specialize in only some segments to take advantage of their expertise and experience and avoid complicated management [54]. A concentration strategy may improve customer screening and monitoring, significantly supporting banks’ lending activities [55]. When these upsides of bank specialization are in place, it is less likely for concentrated banks to effectively confront the pressure of competition.

To test the moderating role of bank diversification, we extend the baseline model as follows:

$$Y_{i,t}\;=\;\alpha_{0}\;+\;\alpha_{1}\;\times \;Y_{i,t-1}\;+\;\alpha_{2}\;\times \;COMP_{t-1}\;+\;\alpha_{3}\;\times \;COMP_{t-1}\;\times \;DIVER_{i,t-1}\;+\;\alpha_{4}\;\times \;X_{i,t-1}\;+\;\alpha_{5}\;\times \;Z_{t-1}\;+\;v_{i}\;+\;\varepsilon_{i,t}$$ (7)

where the coefficient on the interaction term *COMP* × *DIVER* reveals whether and how the impact of bank competition on the quantity and quality of bank credit alters according to bank diversification. As consistently displayed in the paper, we capture bank diversification by the share of non-interest income in operating income.

We now discuss the results for the impact of diversification on the association between competition and lending volume (Table 10)/credit risk (Table 11). As a reminder, the results still show that our findings for the standalone impact of bank competition on two dimensions of bank lending still hold for the expanded regressions. These results once again confirm that banks increase their loan volume less aggressively and suffer more credit risk due to greater competition.

**Table 10:** Impact of diversification on the competition-lending volume nexus.

	Dependent variable: Loan growth rate					
	(1)	(2)	(3)	(4)	(5)	(6)
Lagged dependent variable	-0.142***	-0.202***	-0.258***	-0.203***	-0.268***	-0.304***
	(0.022)	(0.033)	(0.038)	(0.020)	(0.057)	(0.058)
Lerner	92.405***					
	(23.394)					
Lerner*Diversification	-132.397***					
	(36.969)					
CR5		246.451***				
		(49.564)				
CR5*Diversification		-170.631***				
		(41.948)				
HHI			493.151***			
			(155.651)			
HHI*Diversification			-1,185.851***			
			(196.698)			
Boone				2,214.515**		
				(1,020.166)		
Boone*Diversification				-998.490***		
				(290.316)		
H1					-119.915***	
					(26.430)	
H1*Diversification					346.400***	
					(33.242)	
H2						-123.995***
						(26.354)
H2*Diversification						304.543***
						(29.289)
Diversification	-52.500***	-15.068	-4.271	-9.819	-48.002***	-50.306***
	(9.334)	(14.687)	(13.964)	(13.686)	(16.216)	(15.405)
Bank controls	Yes	Yes	Yes	Yes	Yes	Yes
Macro controls	Yes	Yes	Yes	Yes	Yes	Yes
Observations	401	401	401	401	401	401
Banks	30	30	30	30	30	30
Instruments	29	29	29	29	29	29
AR(1) test	0.002	0.002	0.003	0.000	0.002	0.002
AR(2) test	0.318	0.117	0.175	0.345	0.297	0.230
Hansen test	0.139	0.155	0.121	0.204	0.147	0.198

Note: The sample data run from 2007 to 2021. All regressions are obtained from the system GMM estimator. Robust standard errors are shown in brackets. Diagnostic tests are reports with p-values. (***, **) establish the significance levels at 1% and 5%.

**Table 11:** Impact of diversification on the competition-lending quality nexus.

	Dependent variable: Loan loss provisions					
	(1)	(2)	(3)	(4)	(5)	(6)
Lagged dependent variable	0.309***	0.523***	0.509***	0.328***	0.427***	0.435***
	(0.061)	(0.060)	(0.060)	(0.058)	(0.061)	(0.066)
Lerner	-1.348***					
	(0.308)					
Lerner*Diversification	0.340***					
	(0.106)					
CR5		-3.027***				
		(0.642)				
CR5*Diversification		1.748*				
		(0.920)				
HHI			-8.679***			
			(1.840)			
HHI*Diversification			7.478*			
			(4.277)			
Boone				-39.070***		
				(13.214)		
Boone*Diversification				1.149***		
				(0.267)		
H1					0.756**	
					(0.306)	
H1*Diversification					-2.490**	
					(1.147)	
H2						0.805***
						(0.304)
H2*Diversification						-2.067**
						(1.031)
Diversification	1.043***	1.510***	1.537***	1.745***	0.014	0.064
	(0.306)	(0.496)	(0.497)	(0.371)	(0.617)	(0.589)
Bank controls	Yes	Yes	Yes	Yes	Yes	Yes
Macro controls	Yes	Yes	Yes	Yes	Yes	Yes
Observations	409	409	409	409	409	409
Banks	30	30	30	30	30	30
Instruments	29	29	29	29	29	29
AR(1) test	0.000	0.000	0.000	0.001	0.003	0.003
AR(2) test	0.412	0.315	0.137	0.145	0.119	0.307
Hansen test	0.134	0.234	0.136	0.115	0.149	0.214

Note: The sample data run from 2007 to 2021. All regressions are obtained from the system GMM estimator. Robust standard errors are shown in brackets. Diagnostic tests are reports with p-values. (***, **, *) establish the significance levels at 1%, 5%, and 10%.

Table 10 shows that the interaction between competition and diversification is significantly positive when using the H-statistic indexes and significantly negative for all other market structure measures. Consistently, the sign of all interaction terms is opposite to that of standalone competition measures, thereby suggesting that more diversified banks are better positioned to protect their loan growth under greater competition pressure. Similarly, for the estimates reported in Table 11, we have appropriate evidence to suggest that while bank competition may raise the risk of credit portfolios, the level of risk may be mitigated if banks can protect their lending activities by increasing their reliance on multiple banking activities. Our results reveal that income diversification could act as an effective risk absorber since it could mitigate the adverse effect of bank competition on credit risk.

Table 12 employs an alternative measure of income diversification via the Herfindahl-Hirschman approach that considers all sources of bank income, including net interest income, commissions and fees, net gains from trading activities, and other non-lending-based income. Similar to all results discussed above, we find evidence that the detrimental impact of market competition on bank lending (reduced loan volume and increased credit risk) is less pronounced in banks with higher income diversification.

**Table 12:** Alternative measure of bank diversification.

	Dependent variable: Loan growth rate						Dependent variable: Loan loss provisions					
	(1)	(2)	(3)	(4)	(5)	(6)	(7)	(8)	(9)	(10)	(11)	(12)
	Lerner	CR5	HHI	Boone	H1	H2	Lerner	CR5	HHI	Boone	H1	H2
Lagged dependent variable	-0.158***	-0.114***	-0.222***	-0.108***	-0.397***	-0.407***	0.425***	0.641***	0.528***	0.394***	0.395***	0.546***
	(0.030)	(0.036)	(0.034)	(0.027)	(0.057)	(0.052)	(0.040)	(0.053)	(0.045)	(0.050)	(0.070)	(0.048)
Competition	63.327**	290.574***	700.457***	2,176.532***	-124.113***	-121.243***	-0.786**	-3.860***	-4.972***	-38.051***	0.787**	1.893***
	(30.713)	(49.125)	(142.757)	(592.190)	(21.739)	(21.448)	(0.358)	(0.537)	(1.733)	(10.642)	(0.344)	(0.274)
Competition*DiversificationHHI	-123.217***	-8.595**	-281.033**	-395.233***	197.825***	156.139***	0.491	1.661***	9.224***	9.217**	-2.086**	-1.288
	(16.345)	(3.534)	(143.172)	(135.899)	(27.119)	(20.227)	(0.952)	(0.414)	(2.987)	(4.636)	(1.036)	(0.895)
DiversificationHHI	-17.644***	-6.348	4.638	9.470	-26.892***	-32.200***	0.529***	0.465	0.858***	1.381***	0.604**	0.669**
	(6.105)	(7.749)	(7.219)	(9.192)	(8.501)	(6.110)	(0.192)	(0.315)	(0.294)	(0.319)	(0.268)	(0.264)
Bank controls	Yes	Yes	Yes	Yes	Yes	Yes	Yes	Yes	Yes	Yes	Yes	Yes
Macro controls	Yes	Yes	Yes	Yes	Yes	Yes	Yes	Yes	Yes	Yes	Yes	Yes
Observations	401	401	401	401	401	401	409	409	409	409	409	409
Banks	30	30	30	30	30	30	30	30	30	30	30	30
Instruments	29	29	29	29	29	29	29	29	29	29	29	29
AR(1) test	0.000	0.000	0.000	0.000	0.000	0.000	0.002	0.000	0.003	0.001	0.000	0.003
AR(2) test	0.305	0.292	0.224	0.196	0.204	0.390	0.363	0.354	0.114	0.411	0.237	0.208
Hansen test	0.185	0.126	0.130	0.227	0.127	0.206	0.144	0.180	0.144	0.128	0.151	0.223

Note: The sample data run from 2007 to 2021. All regressions are obtained from the system GMM estimator. Robust standard errors are shown in brackets. Diagnostic tests are reports with p-values. (***, **) establish the significance levels at 1% and 5%.

## Conclusions

This paper extends the existing literature by examining an important channel through which bank competition could drive the real economy by comprehensively influencing bank lending. We build a series of different structural (concentration indicators) and non-structural (Lerner, Boone, and Panzar-Rose H-statistic indexes) competition measures, given the stylized fact that the reliance on solely one individual measure could bring about a misleading conclusion. Analyzing a sample of commercial banks during 2007–2021 in a single Vietnamese banking market, we find various interesting results that could be summarized as follows.

First, banks tend to slower their loan growth in the context of high competition, and this linkage firmly holds across both structural and non-structural competition measures. Second, greater competition may cause banks to suffer from more credit risk, and the effect is consistently validated by all different market structure measures. Third, with respect to the association between bank competition and the price of credit, our empirical evidence is conflicting based on different measures to analyze the banking market structure. Overall, a competitive banking sector may result in tremendous drawbacks for the economy, in the form of decreased credit quantity and deteriorated credit quality, while potential benefits to society in terms of lower prices as widely suggested by the theoretical literature are not confirmed according to mixed findings obtained. We demonstrate that our findings are robust as various robustness tests are performed, including employing alternative measures of bank lending dimensions and bank market structure, removing the periods of the 2007–2009 financial crisis and the 2020–2021 COVID-19 pandemic, and changing the empirical estimations technique. In addition, our deeper analysis reveals that the adverse impact of banking competition on the quantity and quality of bank credit is less significant for banks with a higher degree of income diversification, i.e., banks that rely more on non-traditional segments rather than lending and banks that diversify across various business lines. This result suggests that bank diversification may protect the quantity and quality of bank lending from the detrimental impact of bank competition.

From a perspective of policy implications, our findings on the adverse effect of bank competition on bank loan growth and credit risk may raise concerns among regulators about competition in the banking market. Given more lending problems in the context of greater competition, they have to take necessary actions on competition policies in the banking market or they should require banks operating in highly competitive environments to intensify their screening and monitoring activities. In addition, this study offers valuable insights for bank managers and banking regulators about the significance of diversification in the linkage between competition and lending. Accordingly, they could suggest business strategies and rigid policies to encourage diversification so that banks can protect the development of bank lending in quantity and quality. For a research implication, multiple measures of bank market structure should be used simultaneously since each of them looks into one particular aspect of bank competition. Scholars need to be aware of their analysis shortcomings if they fail to allow for many additional competition measures in their work.

## Supporting information

S1 Data (XLSX)
